# Evaluation of Attenuation of Lumbar Epaxial Musculature in Dogs with Spinal Pathology

**DOI:** 10.3390/ani15101468

**Published:** 2025-05-19

**Authors:** Robert Cristian Purdoiu, Ionuț Claudiu Voiculeț, Joana Alexandra Aldea, Radu Lăcătuș, Teodora Patrichi, Felix Daniel Lucaci, Tatjana Chan, Patrick Kircher, Sorin Marian Mârza

**Affiliations:** 1 Laboratory of Radiology and Medical Imaging, Faculty of Veterinary Medicine, University of Agricultural Sciences and Veterinary Medicine, Calea Mănăștur 3-5, 400372 Cluj-Napoca, Romania; robert.purdoiu@usamvcluj.ro (R.C.P.); ionut-claudiu.voiculet@usamvcluj.ro (I.C.V.); joana.aldea@student.usamvcluj.ro (J.A.A.); radu.lacatus@usamvcluj.ro (R.L.); sorin.marza@usamvcluj.ro (S.M.M.); 2 Clinic for Diagnostic Imaging, Department of Diagnostics and Clinical Services, Vetsuisse Faculty, University of Zurich, Winterthurerstrasse 260, 8057 Zurich, Switzerland; tatjana.chan@uzh.ch (T.C.); patrick.kircher@uzh.ch (P.K.)

**Keywords:** computed tomography (CT), canine spine, epaxial muscles, Hounsfield unit (HU), muscle attenuation, myosteatosis, intervertebral disc disease (IVDD), spinal cord injury

## Abstract

This study evaluated attenuation values of lumbar epaxial musculature in dogs with acute spinal pathology using computed tomography (CT) and compared them with values in dogs without spinal disease. Sixty client-owned dogs were included: thirty dogs with thoracolumbar spinal lesions (intervertebral disc herniation) and thirty control dogs without spinal abnormalities. Mean Hounsfield unit (HU) values of epaxial muscles (multifidus–longissimus–iliocostalis group) were measured bilaterally at three lumbar levels for each dog—one level cranial to the lesion, the lesion level, and one level caudal to the lesion; for controls, the corresponding segments were T13, L1, L2, and L3. Dogs with spinal pathology showed a significant local decrease in muscle HU at the lesion site (average ~48 HU) compared to the segment cranial to the lesion (~50–51 HU, *p* < 0.01). In contrast, control dogs had relatively uniform muscle HU (~52–54 HU) across T13–L3 with no significant differences between these levels. Side to side differences were minimal in both groups. A logistic regression using the HU drop between segments correctly classified ~70% of cases, indicating moderate diagnostic value. Age and breed influenced overall muscle HU; older dogs had lower values (r = –0.39, *p* = 0.03 in controls), and French Bulldogs showed lower HU than other breeds. In conclusion, dogs with acute thoracolumbar disc herniation exhibit a focal reduction in paraspinal muscle HU at the lesion level. This acute change is subtle but detectable with CT and may serve as an additional indicator of lesion presence or chronicity, although its clinical utility requires further investigation in larger studies.

## Simple Summary

Computed tomography (CT) can quantify muscle composition in dogs by measuring tissue density in Hounsfield units (HU). Lower HU indicates fattier or less dense muscle. This study compared lumbar epaxial muscle attenuation between dogs with thoracolumbar spinal disc herniations to that of healthy control dogs. Dogs with spinal injuries had lower muscle HU at the site of the lesion (suggesting acute muscle degeneration) compared to controls, whereas their muscle HU farther away from the lesion remained closer to normal. Control dogs had uniform muscle HU along the spine. We also noted that older dogs and certain breeds (like French Bulldogs) tended to have lower muscle HU. These findings suggest that CT assessment of paraspinal muscles may provide additional information about the presence and duration of spinal conditions in dogs.

## 1. Introduction

Computed tomography (CT) provides an objective means of evaluating skeletal muscle composition by measuring X-ray attenuation in Hounsfield units (HU). Lower HU values in muscle indicate reduced tissue density, often due to increased intramuscular fat or fibrous infiltration—a condition termed myosteatosis [1]. In human medicine, decreased paraspinal muscle attenuation has been correlated with age-related sarcopenia and chronic spinal disorders. For example, patients with lumbar spine degeneration or chronic back pain tend to exhibit lower paraspinal muscle HU values, reflecting fatty degeneration of muscle tissue [2,3,4]. Hwang et al. (2021) [2] reported that intervertebral disc pathology was associated with reduced attenuation of the adjacent paraspinal muscles in human patients. Such changes in muscle quality have important implications, as diminished muscle density is linked to poorer functional outcomes and increased risk of comorbidities in humans [3]. These insights have prompted interest in applying similar assessments in veterinary patients.

Recent studies suggest that CT-measured muscle attenuation can be a valuable indicator of muscle condition in dogs, as well. Boström et al. (2018) demonstrated the feasibility and reliability of quantifying epaxial muscle cross-sectional area and density in dogs using CT (and MRI), reporting high agreement between the modalities. Their work, focused on Dachshunds, also emphasized that increased fat infiltration within muscle correlates with diminished contractile function [5]. Another study by Sutherland-Smith et al. (2019) compared epaxial muscle attenuation in young versus old dogs and found that older dogs had significantly lower HU values than younger dogs, consistent with an age-related decline in muscle quality [6]. Lee et al. (2023) further confirmed in a large cohort of small-breed dogs that advancing age is accompanied by decreased epaxial muscle HU and muscle cross-sectional area, indicative of sarcopenia and fatty muscle replacement [7]. These veterinary studies mirror findings in humans, reinforcing that muscle attenuation is sensitive to degenerative changes due to aging or disuse.

Despite these developments, the impact of spinal pathology itself on epaxial muscle attenuation in dogs remains incompletely characterized. In humans, localized spinal lesions (such as intervertebral disc herniation or nerve root compression) can lead to atrophy and fatty infiltration of adjacent musculature [2]. Thoracolumbar intervertebral disc extrusion is one of the most common neurologic injuries in dogs, often leading to acute spinal cord compression and paralysis [5]. It is hypothesized that a similar process occurs in canine patients with spinal injuries or chronic intervertebral disc disease; the paraspinal (epaxial) muscles at the level of the lesion may undergo disuse atrophy, inflammation, or fat replacement, resulting in lower HU values compared to unaffected dogs. In contrast, muscles further away from the lesion (caudal or cranial) would be less affected. The objective of this study was to determine whether thoracolumbar spinal lesions in dogs are associated with measurable declines in epaxial muscle density. We quantified muscle HU values from CT scans of affected dogs and compared them to values from a control group of dogs without spinal disease. We further examined differences between body sides (left vs. right symmetry), variation with location relative to the lesion, and correlations with age, sex, and breed. We hypothesized that dogs with acute spinal lesions would show a localized decrease in muscle HU at the lesion site compared to controls, while muscles further away would remain unaffected. We aimed to determine whether spinal lesions are associated with measurable declines in muscle density and assess the potential utility of this CT-based evaluation as a tool for identifying or characterizing spinal injury in canine patients.

## 2. Materials and Methods

### 2.1. Animals

The study evaluated a total of 60 client-owned dogs, comprising 30 dogs with spinal pathology (the “patient group”) and 30 healthy dogs without evidence of spinal disease (the “control” group). Dogs of various breeds, both genders, and ages ranging from 1 to 10 years (mean age ~3.8 years in the patient group, ~5.7 years in the control group) were included. The patient group consisted of dogs diagnosed with acute thoracolumbar spinal lesions (intervertebral disc herniations with spinal cord compression, located between vertebrae T13 and L3) confirmed via diagnostic imaging. These dogs presented with clinical signs of spinal injury (e.g., pain, paresis, or paralysis, onset of symptoms 1–7 days before clinical evaluation, imaging, and treatment). Breeds represented included French Bulldog (n = 9), Dachshund (n = 5) Bichon Frisé (n = 4), mixed breed (n = 6), and others (n = 6), with brachycephalic and chondrodystrophic breeds notably common. The spinal pathology group included 18 males and 12 females (male:female ratio 60:40). The control group comprised 16 males and 14 females (male:female ratio 53:47), with a similar age range (1–10 years). Control dogs were client-owned pets that underwent CT scans for reasons unrelated to spinal disease (e.g., trauma screening, gastrointestinal pathology, lung disease, or oncology cases) and were confirmed to have no spinal abnormalities clinically or on imaging. Ethical review and approval were waived for this study, as it was a retrospective analysis of computed tomography (CT) scans obtained from client-owned dogs during routine clinical evaluations at the Radiology and Imaging Service of the Faculty of Veterinary Medicine, University of Agricultural Sciences and Veterinary Medicine, Cluj-Napoca, between 2022 and 2024. The study utilized existing diagnostic imaging data collected for clinical purposes, and no experimental procedures or interventions were performed on the animals specifically for this research. Owner consent was obtained for the use of the imaging data, and all procedures adhered to the ethical guidelines for animal research. Consequently, formal approval from an Institutional Review Board or Ethics Committee was not required for this retrospective study.

### 2.2. Imaging and Measurement Protocol

Computed tomography (CT) scans of the thoracolumbar spine were conducted using a multi-slice CT scanner (Siemens Somatom Scope, 120 kVp, 110 mAs, 512 × 512 matrix, soft tissue and bone kernel, 1.5 mm slice thickness, pitch of 1). Transverse (axial) CT images were reconstructed using bone or soft tissue algorithms suitable for muscle evaluation. Three axial images per dog were analyzed. In the control group, the images were collected at the T13–L1 level because the region is more susceptible to thoraco-lumbar disc pathology [8,9,10,11].

- Cranial adjacent level (M1): the transverse image one vertebra cranial to the lesion (for controls, the first lumbar vertebra, L1).
- Lesion level (M2): the transverse image through the center of the identified spinal lesion (for controls, an analogously “middle” level—we used the second lumbar vertebra, L2, as the lesion-level reference in controls).
- Caudal adjacent level (M3): the transverse image one vertebra caudal to the lesion (for controls, the third lumbar vertebra, L3).

These three levels enabled us to sample muscle attenuation immediately above, at, and below the lesion. In control dogs, which have no lesion, we selected L1, L2, and L3 as corresponding cranial, middle, and caudal segmental levels, respectively, for consistency. (This region was chosen as it is commonly affected in disc disease cases.)

**Figure 1:**
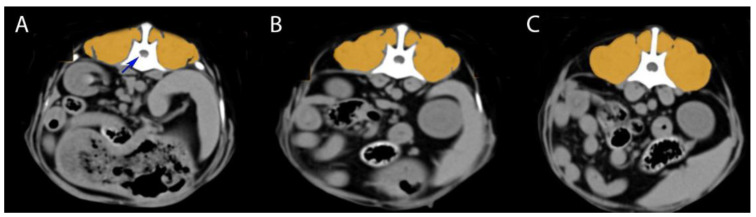
Measurement technique on a representative case—CT transverse images from a Bichon Frisé with an acute L1–L2 disc herniation (blue arrow indicates herniated disc material compressing the spinal cord). The epaxial muscles are outlined in orange. (**A**) Measurement at the herniation level (L1–L2); (**B**) measurement one segment cranial to the herniation (T13–L1 level); (**C**) measurement one segment caudal to the herniation (L2–L3 level).

Epaxial muscle groups (multifidus, longissimus, iliocostalis muscles) were identified bilaterally on each image. Using RadiAnt DICOM viewer 2025.1 (Poznań, Poland), Horos DICOM viewer (Annapolis, MD, USA), the muscle area was manually outlined through freehand tracing, excluding adjacent fat or tissues. The software calculated the mean muscle attenuation (HU), standard deviation, and cross-sectional area. Six HU readings per dog were collected; this process was performed bilaterally on each image (left and right sides were measured separately) to assess any asymmetry. In total, six HU readings were obtained per dog (left and right at M1, M2, and M3). All measurements were performed by a single trained observer to ensure consistency. Figure 1 illustrates the measurement technique on a representative case.

### 2.3. Data Analysis and Statistical Methods

Collected HU values were compiled for statistical analysis. We first performed intragroup comparisons examining differences in muscle HU across the three measured locations (M1, M2, M3) within each group using appropriate non-parametric tests (Mann–Whitney U tests for independent samples). Differences across the three measured levels within each group were assessed using a Friedman test for the patient group (which had non-normally distributed repeated measures) and a repeated-measures ANOVA for the control group (which met assumptions for parametric testing). Where significant overall differences were found across levels, pairwise comparisons were made with paired *t*-tests or Wilcoxon signed-rank tests, as appropriate.

Side to side (left vs. right) comparisons within each group to detect any lateral asymmetry were evaluated with paired tests (paired *t*-tests or Wilcoxon, as appropriate per normality). In addition, we calculated the Pearson correlation between left and right values across dogs to assess overall symmetry.

Correlations between muscle HU and age were assessed using Pearson’s correlation coefficient (and Spearman’s rank for confirmation).

Differences by sex were analyzed using independent-sample Mann–Whitney U tests.

Breed differences were explored using a Kruskal–Wallis test (given multiple breed categories), followed by Dunn’s post hoc tests for pairwise breed comparisons. Because the number of dogs per breed was small in some cases, these breed comparisons were considered exploratory.

A multivariate analysis (multiple linear regression) was performed to model muscle HU as a function of age, sex, and group (patient vs. control), and logistic regression was used to predict patient vs. control status from the HU measurements. Statistical significance was set at *p* < 0.05 for all analyses. Data analysis was conducted using IBM SPSS Statistics 29 software (IBM Corp., Armonk, NY, USA).

## 3. Results

### 3.1. Intragroup Analysis (Segmental Differences Within Each Group)

All 60 dogs (30 patients and 30 controls) underwent successful CT measurement of epaxial muscle attenuation. The quantitative data confirmed a high degree of bilateral symmetry in muscle attenuation overall.

Patient Group (Dogs With Spinal Lesions): In dogs with spinal lesions (n = 30), epaxial muscle HU varied across the three measured locations (segments) on each side (M1 cranial, M2 lesion-level, and M3 caudal). On the left side, the mean HU of the epaxial muscles declined from 50.8 ± 6.1 at the segment cranial to the lesion (M1) to 48.2 ± 6.7 at the lesion level (M2) and 48.1 ± 8.0 at the segment caudal to the lesion (M3). Statistical analysis confirmed that left-side epaxial muscle HU at the lesion (M2) and post-lesion levels (M3) were significantly lower than at the pre-lesion level (M1) (*p* = 0.001 and *p* = 0.027, respectively). The difference between the at-lesion (M2) and post-lesion (M3) levels on the left was not significant. The right side showed a similar pattern, as mean HU values declined from 50.4 ± 7.3 pre-lesion (M1) to 48.1 ± 7.3 at the lesion (M2) and 47.8 ± 7.8 post-lesion (M3). This decline on the right was significant from pre-lesion (M1) to at-lesion (M2) (*p* = 0.001). The pre-(M1) vs. post-lesion (M3) difference on the right side showed a borderline trend (*p* = 0.055) but did not reach significance at the 5% level, and there was no significant difference between the at-lesion (M2) and post-lesion segments (M3) (*p* > 0.7). Left vs. right side differences at each corresponding location were minimal and not statistically significant (all *p* > 0.4), indicating no strong lateral asymmetry in muscle attenuation in the patient group. For example, at the lesion level M2, left mean = 48.2 vs. right mean = 48.1. The Pearson correlation between left and right HU values across patients was high (r ≈ 0.9), underscoring bilateral symmetry. Thus, despite a few unilateral lesions, on average, there was no strong lateral asymmetry in muscle attenuation in this acute patient cohort.

Control Group (Dogs Without Spinal Pathology): In dogs without spinal pathology (n = 30), epaxial muscle HU was measured at three corresponding lumbar levels (L1, L2, and L3). No significant differences were found among L1, L2, and L3 HU values on the same side. On the left side, mean HU values were 53.4 ± 7.3 at L1, 54.2 ± 7.5 at L2, and 52.9 ± 8.0 at L3, with no significant variation across these levels (*p* = 0.20, Friedman test). Similarly, on the right side, mean HU values were 52.7 ± 8.0 (L1), 52.4 ± 7.6 (L2), and 51.8 ± 7.7 (L3), again showing no statistically significant differences (*p* = 0.29). Thus, the epaxial musculature in healthy dogs appeared uniform in density along the examined lumbar segments. A minor side difference was noted at the L2 level, as the left side HU was slightly higher than that of the right side (54.2 vs. 52.4, *p* = 0.008), but, overall, left–right symmetry was preserved in controls.

**Figure 2:**
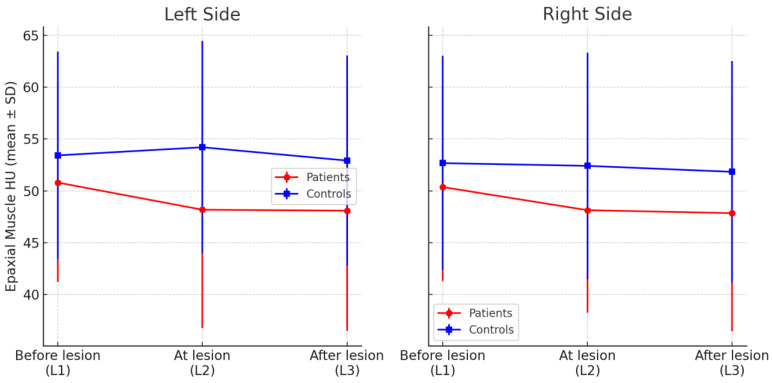
Mean Hounsfield unit (HU) values of the epaxial muscles for patients (red circles) and controls (blue squares) shown across the three segment locations on the left and right sides (error bars = SD). Patients exhibit a drop in muscle HU at the lesion level, whereas controls show relatively stable muscle HU across L1–L3.

Table 1 and Figure 2 (below) presents the mean HU ± SD for each group at each segment and side. Within the patient group, HU at the lesion and post-lesion segments was significantly lower than at the pre-lesion segment on each side (*p* < 0.05). No significant HU differences were observed among segments in the controls (*p* > 0.1). Consistent with the text above, left–right differences were not significant in patients; in controls, only a small left > right difference at L2 was noted (*p* < 0.01).

### 3.2. Intergroup Comparison (Patients vs. Controls)

Comparing the two groups at corresponding segment levels revealed generally lower muscle HU in the dogs with spinal pathology, especially at and beyond the lesion site (M2, M3). At the segment one level cranial to the lesion (patients’ “before lesion”M1 vs. control L1), there was a slight difference in mean HU (patients lower by ~2–3 HU on each side), but it was not statistically significant (e.g., left side 50.8 vs. 53.4, *p* = 0.30; right side 50.4 vs. 52.7, *p* = 0.36).

At the lesion level (patient M2 vs. control L2), a significant difference emerged on the left side, as patients’ left-side HU at the lesion (M2) averaged 48.2 ± 6.7 compared to 54.2 ± 7.5 in controls at the analogous level L2 (*p* = 0.036). The right side at the lesion level (M2) showed a similar lower HU in patients (48.1 vs. 52.4), but this difference did not reach significance (*p* = 0.12). For the segment caudal to the lesion (patient M3 vs. control L3), the patients group again had a lower mean HU than the control group (approximately 48.1 vs. 52.9 on the left; 47.8 vs. 51.8 on the right), but these differences were not statistically significant (left *p* = 0.09; right *p* = 0.17).

In summary, dogs with spinal lesions exhibited a localized reduction in epaxial muscle HU relative to healthy dogs—most notably at the lesion level (statistically significant on the left side)—whereas at the segment above the lesion there was little difference between groups. The group difference at one segment caudal was in the expected direction (patients lower than controls) but did not achieve significance in this sample.

When muscle attenuation values were averaged across all three segments for each side, the overall mean HU was slightly lower in the patient group compared to controls. The mean HU for the entire left side was about 49.5 in patients vs. 54.0 in controls (difference ~4.5 HU, *p* = 0.09). For the right side, patients averaged roughly 48.8 vs. 52.3 in controls (difference ~3.5 HU, *p* = 0.18). These overall group differences did not reach the threshold for statistical significance. Thus, while there was no significant difference in global mean muscle attenuation between dogs with and without spinal pathology, the segment-specific comparisons above highlight localized differences at the lesion level.

### 3.3. Effects of Age, Sex, and Breed

We evaluated whether age, sex, or breed influenced paraspinal muscle attenuation. These results are summarized in Table 2 for clarity.

**Age:** Age proved to be an important factor influencing muscle HU in healthy dogs. In the control group (ages 1–10 years), there was a moderate negative correlation between age and mean epaxial muscle HU (older dogs tended to have lower HU values). Pearson’s correlation coefficient was approximately –0.39, which was statistically significant (*p* = 0.03). Thus, healthy older dogs showed evidence of age-related muscle attenuation decline on CT (consistent with increased intramuscular fat in aging muscle). In the patient group (which was generally younger on average), no significant correlation between age and HU was found (e.g., Pearson r ≈ –0.19, *p* > 0.3). In other words, among dogs with spinal lesions, muscle HU did not show a clear linear relationship with age in this sample, likely because the effect of the acute lesion (and variability between individuals) overshadowed the more subtle age effect. We note that our sample size for this analysis is limited (the oldest control was 10 years old, so we lacked truly geriatric dogs), and a post hoc power analysis indicates that detecting small age effects would require more animals. Nonetheless, the finding in the controls aligns with prior studies that have documented age-related muscle changes.

**Sex:** Sex alone did not show a significant association with muscle attenuation in this dataset. In the control group, females had a slightly lower mean HU than males (overall mean HU in females was about 5 HU lower than in males), but this difference was only a non-significant trend (*p* = 0.08). In the patient group, there was virtually no difference in HU between males and females (male mean 49.9 vs. female 47.4, *p* = 0.49). Thus, no conclusive effect of sex on epaxial muscle HU was demonstrable. Any minor sex-related differences in muscle density were overshadowed by inter-individual variability and other factors, such as age or breed. A difference of ~5 HU had only around 50–60% power, so it is possible that a subtle true sex difference exists (with males perhaps being a bit more muscular), but it could not be confirmed here. For now, we interpret that sex is not a major determinant of paraspinal muscle HU compared to factors like disease or age.

**Breed:** Breed-related differences in muscle attenuation were observed, although evaluating these was outside of the primary aim of the study. French Bulldogs in our sample (n = 9, all in the patient group) had the lowest epaxial muscle HU values among the breeds analyzed, with an average of 48.3 ± 5.1 HU. This was significantly lower than the mean HU for Bichon Frisé (n = 5, 53.8 ± 3.9 HU, *p* = 0.03) and for mixed-breed dogs (n = 6, 52.1 ± 4.7 HU, *p* = 0.02). Dachshunds and several other small breed dogs exhibited intermediate HU values (e.g., Dachshund patients had HU in the low 50s on average), but the sample sizes for these breeds were limited, making firm conclusions difficult. Notably, none of the French Bulldogs were represented in the control group, so we could not establish a baseline HU for healthy individuals of this breed [9,10,12]. Overall, these findings suggest that certain breeds, particularly chondrodystrophic breeds, like French Bulldogs, may have inherently lower epaxial muscle HU or greater susceptibility to low muscle HU when affected by spinal disease. However, given the small numbers, further investigation with larger breed-specific samples is necessary to disentangle genetic or conformational influences on muscle density from the changes caused by spinal pathology.

Table 2 provides a summary of these secondary analyses (age, sex, breed) with the observed values and significance.

### 3.4. Predictive Modeling of Pathology Status

To explore the diagnostic value of the muscle attenuation measurements, a model was developed to predict the presence of spinal pathology (patient vs. control status) from the HU data. A logistic regression classifier was trained using each dog’s three segmental HU measurements per side (M1, M2, and M3 for patients; L1, L2, and L3 for controls), along with age and sex as predictors. Given the limited sample size (30 affected + 30 controls) and the inter-correlation of the HU features, the model’s performance was modest. Using five-fold cross-validation, the logistic model achieved an average accuracy of approximately 70% in distinguishing dogs with spinal lesions from controls. This corresponded to a sensitivity of about 73% and specificity of about 67% (i.e., it correctly identified roughly 73% of the patients and 67% of the controls in cross-validated predictions). For comparison, a non-linear classifier (random forest) yielded a similar accuracy of ~73%, indicating no major gain from more complex modeling on this dataset.

The behavior of the logistic model highlighted certain features as most informative for prediction. It assigned the greatest weight to the difference in muscle HU between the cranial segment and the lesion segment. In other words, a larger drop in HU from M1 to M2 strongly pushed the model towards predicting “pathology.” Indeed, in our data, about 77% of the dogs in the patient group had a measurable decline in HU from the cranial adjacent segment to the lesion level compared to only ~50% of the control dogs (some control dogs had minor declines from L1 to L2, but many had an increase or stayed the same). Consistent with this, the absolute HU value specifically at the lesion level (M2) was also an important predictor in the model. On the other hand, the HU values at the far cranial (M1) or far caudal (M3) segments, as well as the overall mean HU, were less informative in the multivariable context. This supports the notion that it is the localized change (drop) that signals pathology, rather than a generally low HU.

Age was included in the model and had a minor effect, as older age slightly increased the odds of being classified as “pathology,” but this likely reflects that older dogs in our sample were more often controls with age-related low HU, which could confuse the model. Sex was not a significant contributor.

In summary, predictive modeling reinforced our main finding that focal HU decrease at the lesion site (relative to adjacent areas or normal dogs) is the key identifier of spinal pathology. While 70% accuracy is not high enough for clinical diagnosis on its own, it is better than chance and suggests that with further refinement (or as part of a combined diagnostic approach), CT muscle attenuation could aid in detecting or confirming spinal problems. The model also underscores that a dog with a notably low HU at one vertebral level compared to the next is likely to have a lesion at that level.

## 4. Discussion

This study provides new insights into how thoracolumbar spinal pathologies affect the quality of adjacent musculature in dogs. The key finding is that dogs with spinal disc herniations exhibit localized reductions in epaxial muscle attenuation at the level of the lesion, even though their overall muscle density may remain comparable to that of healthy dogs. In other words, rather than causing uniform wasting of the back muscles, a focal spinal injury tends to produce focal myopathy—a decrease in muscle HU confined to the region of the lesion—while muscles further away from the lesion continue to resemble those of normal dogs. This interpretation is supported by our statistical analyses, as there was no significant difference in the global mean HU between the patient and control groups, but a significant effect emerged when comparing specific positions within the patients (pre-lesion vs. lesion-level segments). Healthy dogs showed consistent muscle HU across the lumbar levels (anatomical homogeneity), whereas diseased dogs showed a marked decrease at the lesion site. Clinically, this suggests that when evaluating a dog with a spinal injury, one should focus on the muscles immediately adjacent to the lesion rather than expect a generalized change in muscle quality throughout the spine. Our logistic regression further supported this by revealing that the presence of a localized HU drop was a strong discriminator between affected and normal dogs. This focal nature of muscular change is important, as it implies that we are detecting the direct effect of the local neural injury (denervation or disuse atrophy) rather than a systemic condition. In practical terms, spinal injuries in these dogs caused targeted muscle deterioration at the lesion level rather than widespread muscle wasting, which aligns with clinical observations that focal nerve compressions or lesions lead to regional muscle atrophy [13,14,15,16].

Findings in human medicine provide context for these results. In human patients, multifidus muscle atrophy and fatty infiltration have been well-documented at the site of chronic lumbar spine lesions or pain generators, often without generalized sarcopenia elsewhere. Hwang et al. (2021) specifically reported lower paraspinal muscle HU in patients with intervertebral disc pathology [2], which is consistent with the lesion-level attenuation decreases observed in our canine patients. We also found that the attenuation deficit was less pronounced one vertebral segment away from the lesion, indicating that the muscle changes are highly localized. This pattern is consistent with the idea that a direct neural injury, localized inflammation, or disuse at a lesion causes regional muscle changes (acute stages may involve edema, and chronic stages progress to fat and fibrous tissue replacement) rather than systemic myopathy. Interestingly, Hwang et al. also noted that CT and MRI were comparably reliable in assessing paraspinal muscle quality [2]. This agrees with the findings of Boström et al. in dogs, which showed that CT is an efficient and reliable method for evaluating epaxial muscles [5]. Together, these points suggest that CT can stand in for MRI in quantifying muscle degeneration, allowing for broader clinical application of such assessments.

Our data did not show significant differences beyond one segment away, supporting the notion that the effect is segmental. The likely explanation is the segmental innervation of epaxial muscles; for example, a disc herniation at L1–L2 primarily affects the muscles innervated by the L1–L2 spinal nerves. Muscles at L3–L4 would be innervated by L3–L4 nerves, which remain intact in that scenario, so one would not expect an acute change there (aside from generalized disuse due to pain). Additionally, when one segment’s muscles are weakened, other segments’ muscles may partially compensate to maintain posture, which could mitigate more widespread atrophy.

Another important aspect of our results is the strong bilateral symmetry observed in muscle attenuation. Even in dogs with unilateral neurologic deficits (e.g., one-sided radiculopathy or lateralized disc herniation), the left–right differences in HU were minimal on average. This suggests that compensatory or cross-innervation effects might preserve muscle on both sides or that systemic factors (such as circulating inflammatory cytokines or reduced overall activity) affect both sides similarly. In agreement with this, previous canine studies did not identify significant side to side discrepancies in epaxial muscle metrics in healthy animals [5], and our study shows that this symmetry largely holds, even in the presence of a focal pathology. From a practical perspective, this means that measuring either side’s epaxial muscles at the lesion level will usually be representative, and one should not necessarily expect drastic unilateral atrophy unless the lesion is highly chronic and specifically affecting nerve roots on one side. When unilateral differences do occur (for instance, a ~2–3 HU lower value on the more affected side), they likely indicate long-standing, severe nerve root compression leading to muscle disuse on that side (as sometimes seen in horses with unilateral back pain, for example) [17,18], but our acute cases did not reach that point. Such findings could potentially help identify the side of a lateralized lesion in some cases, but, in our cohort, the side to side effect was subtle compared to the overall lesion effect.

The influence of age on muscle attenuation in dogs was confirmed in the control group and merits discussion. Sutherland-Smith et al. (2019) reported that older dogs have significantly lower epaxial muscle HU than young dogs, a result mirrored by Lee et al. (2023) in a larger sample [6,7]. Our data showed the same pattern in healthy dogs—a moderate negative correlation between age and HU—reinforcing that age-related muscle degeneration (sarcopenia and myosteatosis) occurs in canines just as it does in humans [19]. Notably, we did not see this correlation in the diseased group. This could be because our patient group’s ages were clustered in middle age (most were ~4–8 years old, with fewer very old dogs) and/or because any age effect was overridden by the severity of the pathology. For example, a young dog with a severe, chronic disc lesion may have very low muscle HU due to disuse atrophy and fat replacement, whereas an older dog with an acute injury might still have relatively higher HU [20,21,22]. Thus, spinal pathology can be a confounding factor that masks the typical age-related trend. Our regression analysis hinted at this, as the age*group interaction term was negative (though not statistically significant in the final model), suggesting that the slope of age vs. HU is more steeply negative for healthy dogs than for dogs with lesions. In practical terms, this indicates that in the context of disease, muscle attenuation is influenced more by lesion chronicity than the dog’s chronological age. Clinicians should therefore be cautious not to attribute a low muscle HU in a spinal patient purely to “old age”, as it may well be a consequence of the lesion and could potentially improve with successful treatment and rehabilitation. We mentioned earlier that the statistical power for detecting age effects in the patient group was low; a larger sample might detect a mild age effect even in patients. Regardless, clinicians should consider age when interpreting HU, as what is “low” for a 2-year-old dog might be normal for a 10-year-old. If possible, comparing against age-matched normal cases would refine the assessment.

The lack of a significant overall sex difference in muscle HU in our study aligns with the limited prior data on sex effects. In human studies, males often have greater muscle mass and sometimes slightly higher muscle attenuation (owing to lower fat content), but results have been mixed [23]. For instance, one study found that healthy men had higher paraspinal muscle volume and HU than women, whereas Hwang et al. (2021) did not find a significant gender difference [2,24,25]. Our canine data hinted at higher HU in males (on average ~5 HU higher than females in controls), but this was not statistically significant. It is possible that with a larger sample or a targeted study, a small sex effect could be demonstrated in dogs, perhaps related to hormonal or body composition differences (males tending to have slightly more lean muscle mass). However, given our current results, sex is not a major determinant of epaxial muscle attenuation relative to the impacts of disease and age.

Breed-related differences, although outside of our primary aim, emerged as a notable factor in muscle attenuation. French Bulldogs showed markedly lower muscle HU values compared to other breeds. This finding can be interpreted in several ways. Frenchies are highly predisposed to vertebral and disc diseases (e.g., hemivertebrae, Type II disc disease) [9,12], and, indeed, many of the French Bulldogs in our sample had significant chronic spinal pathology. It is likely that their low HU readings reflect the severity and chronicity of their conditions (i.e., extensive fatty infiltration and muscle degeneration). It is also conceivable that French Bulldogs—being a chondrodystrophic, brachycephalic breed—might have different baseline muscle composition, possibly with more intramuscular fat even when healthy (for example, due to genetic factors or their often stocky, compact build). To clarify this, data from healthy French Bulldogs would be needed, which we did not have in our control group. Dachshunds and other chondrodystrophic breeds also commonly suffer from intervertebral disc extrusion and related spinal issues [10,12,26]. While our numbers for these breeds were too low to draw firm conclusions, we did observe some Dachshund patients with notably low HU values, as well. The clinical implication is that what constitutes a “normal” HU value might vary by breed. If French Bulldogs generally run lower in muscle HU, using a single universal cutoff might yield false positives for that breed (flagging a normal Frenchie as abnormal). Conversely, a French Bulldog with a long-standing spinal issue might experience an extreme drop in HU (into the 30s or 40s), which should certainly be considered pathological. Breed predispositions also meant that our patient cohort was not demographically balanced relative to the controls, as many controls were mixed-breed or non-chondrodystrophic, whereas the patient group had more high-risk breeds. We acknowledge this as a limitation (selection bias) and suggest that, ideally, future studies should include a broader range of breeds (or focus on one breed at a time) to establish breed-specific reference values.

From a clinical standpoint, measuring the attenuation of epaxial muscles on CT could be a useful adjunct in the assessment of canine spinal disorders. For instance, a dog with chronic back pain might show very low HU values in the paraspinal muscles, supporting a diagnosis of a long-duration process, such as a chronic disc protrusion or spinal arthritis. Improvement in muscle HU over time could potentially serve as a quantitative marker of recovery or successful rehabilitation, as muscle tissue rebuilds and fatty infiltration decreases. In this study, we identified preliminary threshold values (approximately < 33 HU for definitely abnormal muscle and >54 HU for clearly normal muscle) as a starting point for clinicians to interpret muscle attenuation. If a dog’s epaxial muscle HU falls into a “gray zone” between these values, other factors (like age, breed, and overall body condition or activity level) should be considered in interpretation.

The regression model we proposed is an attempt to integrate some of those factors (age, sex) to predict a “normal” expected HU for a given individual. While the specific formula may require refinement with more data, the concept is that veterinarians could input a dog’s characteristics (age, sex, etc.) to obtain an expected muscle HU range—any significant deviation below that might indicate an abnormal degree of muscle fat infiltration for that particular dog. This approach is analogous to using a body condition score or a muscle condition score in practice, but with a numerical, objective metric derived from imaging.

This study has several limitations. The sample size, especially for subgroup analyses, was relatively small, which limits statistical power for subgroup analyses (especially for comparing breeds or sexes). The control dogs were not perfectly matched to the patient dogs in breed composition, as many controls were mixed-breed or non-chondrodystrophic breeds, whereas the patient group had more high-risk breeds. This mismatch could bias some comparisons and make it difficult to separate breed effects from disease effects. The study was retrospective in nature, and the age and sex distributions were not evenly stratified between groups (for example, the patient group skewed younger). Another limitation is that no direct quantification of muscle fat content was performed (e.g., via histopathology or an MRI-based fat fraction measurement). We inferred fat infiltration from low HU values on CT, but one must note that CT attenuation can be influenced by other factors, like edema or mineralization. Nevertheless, given that extremely low HU values (approaching the density of fat) were seen in chronic cases, it is reasonable to attribute those findings to fatty replacement of muscle. We also did not perform follow-up scans of the patient group to monitor the HU of the affected muscles after treatment or over time; a longitudinal study would be very informative to distinguish acute changes (e.g., edema, which might increase muscle water content and could paradoxically raise HU in the short term) from chronic changes (fatty infiltration, which decreases HU). Another limitation is that we analyzed the epaxial muscles as a whole unit and therefore might have missed differences among the individual muscles (multifidus vs. longissimus, etc.). If one muscle atrophied more than the others, our combined ROI would average it out. For simplicity and given the image resolution, we chose the combined approach, but higher-resolution imaging or MRI might separate them. Lastly, the functional status of the muscles (strength or usage) was not directly measured in our patients; we assumed that lower HU implies weaker or more fatty-degenerated muscle, as is generally the case [13,27], but it would be valuable in future work to correlate HU with muscle function tests or the animal’s mobility and clinical outcome.

## 5. Conclusions

In conclusion, this study demonstrates that CT attenuation measurements of the epaxial muscles provide a meaningful reflection of the muscular changes associated with spinal pathology in dogs. Dogs suffering from thoracolumbar disc herniation or intervertebral disc disease (IVDD) tend to have decreased HU values in the epaxial musculature at the lesion site consistent with reduced muscle density due to fat infiltration, edema, or fibrosis. Importantly, these changes are localized—the muscles adjacent to the lesion are the most affected, while more distant muscles may remain near normal. As a result, the overall difference in muscle attenuation between dogs with spinal lesions and healthy dogs is modest, but specific, localized changes are pronounced. We also confirmed that age and breed factors influence muscle attenuation, as older dogs (in the absence of disease) show lower muscle HU (poorer muscle quality) and certain high-risk breeds (e.g., French Bulldogs) may have inherently or pathologically lower values. CT imaging of the spine can thus include an evaluation of the paraspinal muscles, as it can yield supplementary information about the chronicity and severity of a spinal condition. Incorporating HU measurements into routine interpretation could enhance diagnostic assessments; for example, detecting an abnormally low muscle HU in a region might alert the clinician to a chronic process or severe nerve injury that has been present for some time. However, our findings also indicate that the sensitivity and specificity of this measure alone are limited (~70%), so it should complement, not replace, other diagnostic indicators.

Overall, CT-based evaluation of paraspinal muscle attenuation appears to be a useful tool in the context of canine spinal health, especially for research and, potentially, for monitoring. It is non-invasive, adds only a few minutes to image analysis, and provides quantitative data that can be tracked over time. Unlike MRI, CT is widely accessible in veterinary practice; thus, this technique offers a practical alternative for assessing muscle condition when MRI is not available. Therapeutic strategies for spinal disorders (including surgical decompression, anti-inflammatory therapy, and rehabilitation physiotherapy) might benefit from monitoring muscle attenuation as an objective outcome measure, alongside traditional neurological evaluations. The present study establishes preliminary reference values and thresholds for interpreting these measurements. We recommend further studies to expand on these findings—ideally, with larger sample sizes, longitudinal follow-up, and inclusion of diverse breeds and body types—to refine the diagnostic criteria and fully validate the prognostic utility of epaxial muscle attenuation. With continued research, muscle attenuation on CT could become an integrated part of veterinary spinal assessments, helping veterinarians better understand and manage the musculoskeletal consequences of spinal disease in their canine patients. Waived. This study is a retrospective study of non-experimental clinical veterinary practice. 

## Figures and Tables

**Table 1:** Epaxial muscle HU (mean ± SD) for dogs with spinal pathology (“Patients”) and for controls. Patients’ values are shown for the segment cranial to the lesion (M1), at the lesion (M2), and caudal to the lesion (M3). Control values are shown for the analogous segments L1, L2, L3.

Group	Segment	Left HU (Mean ± SD)	Right HU (Mean ± SD)
**Patients**	Before lesion (M1)	50.8 ± 6.1	50.4 ± 7.3
	At lesion (M2)	48.2 ± 6.7 *	48.1 ± 7.3 *
	After lesion (M3)	48.1 ± 8.0 *	47.8 ± 7.8 (^†^)
**Controls**	L1 (proximal segment)	53.4 ± 7.3	52.7 ± 8.0
	L2 (mid segment)	54.2 ± 7.5 ^‡^	52.4 ± 7.6
	L3 (distal segment)	52.9 ± 8.0	51.8 ± 7.7

*Legend:* * Significantly lower than the “Before lesion” (M1 or L1) value on the same side (*p* < 0.05). ^†^ Borderline difference from M1 (*p* = 0.055). ^‡^ Significant side to side difference at this level (left vs. right, *p* < 0.01). No other left–right differences were significant.

**Table 2:** Summary of secondary analyses of epaxial muscle HU by age, sex, and breed. (HU values are averaged per dog across the three segments for comparisons, unless otherwise noted).

Factor (Comparison)	Observation (Mean or Correlation)	*p*-Value
**Age (controls)**	Pearson r = –0.39; older dogs had lower HU	0.03
**Age (spinal patients)**	Pearson r = –0.19; no significant correlation	0.35 (n.s.)
**Sex (controls) (female vs. male)**	Females ~5 HU lower than males (mean HU: F ~50, M ~55)	0.08 (n.s.)
**Sex (patients) (female vs. male)**	Minimal difference (females ~47.4 vs. males ~49.9 HU)	0.49 (n.s.)
**Breed (French Bulldog vs. Bichon)**	French Bulldogs: 48.3 ± 5.1 HU; Bichon Frisé: 53.8 ± 3.9 HU	0.03 *
**Breed (French Bulldog vs. mixed)**	French Bulldogs: 48.3 ± 5.1 HU; mixed-breed: 52.1 ± 4.7 HU	0.02 *
**Breed (other comparisons)**	No other breed differences were statistically significant	n.s.

Note: n.s. = not significant. * = The breed comparisons are from patient group data, except for where breed overlaps with controls. These results should be interpreted cautiously due to small subgroup sizes. A post hoc power analysis indicates that the study had limited power (<0.5) to detect small differences for sex or among most breeds, so only relatively large differences achieved significance.

## Data Availability

The original contributions presented in this study are included in the article. Further inquiries can be directed to the corresponding author(s).

## Abbreviations

The following abbreviations are used in this manuscript:

GIT	Gastrointestinal tract
HU	Hounsfield units
CT	Computed tomography
MRI	Magnetic Resonance Imaging
IVDD	Intervertebral disc disease
